# *Neisseria gonorrhoeae* diagnostic escape from a *gyrA*-based test for ciprofloxacin susceptibility and the effect on zoliflodacin resistance: a bacterial genetics and experimental evolution study

**DOI:** 10.1016/S2666-5247(22)00356-1

**Published:** 2023-02-28

**Authors:** Daniel H F Rubin, Tatum D Mortimer, Yonatan H Grad

**Affiliations:** Department of Immunology and Infectious Diseases, Harvard TH Chan School of Public Health, Boston, MA, USA (D H F Rubin AB, T D Mortimer PhD, Y H Grad MD)

## Abstract

**Background:**

The aetiological bacterial agent of gonorrhoea, *Neisseria gonorrhoeae*, has become resistant to each of the first-line antibiotics used to treat it, including ciprofloxacin. One diagnostic approach to identify ciprofloxacin-susceptible isolates is to determine codon 91 in the gene encoding the A subunit of DNA gyrase, *gyrA*, where coding for the wild-type serine (*gyrA*^91S^) is associated with ciprofloxacin susceptibility and phenylalanine (*gyrA*^91F^) with resistance. The aim of this study was to investigate the possibility of diagnostic escape from *gyrA* susceptibility testing.

**Methods:**

We used bacterial genetics to introduce pairwise substitutions in GyrA positions 91 (S or F) and 95 (D, G, or N), which is a second site in GyrA associated with ciprofloxacin resistance, into five clinical isolates of *N gonorrhoeae*. All five isolates encoded GyrA S91F, an additional substitution in GyrA at position 95, substitutions in ParC that are known to cause an increased minimum inhibitory concentration (MIC) to ciprofloxacin, and GyrB 429D, which is associated with susceptibility to zoliflodacin (a spiropyrimidinetrione-class antibiotic in phase 3 trials for treatment of gonorrhoea). We evolved these isolates to assess for the existence of pathways to ciprofloxacin resistance (MIC ≥1 μg/mL) and measured MICs for ciprofloxacin and zoliflodacin. In parallel, we searched metagenomic data for 11 355 *N gonorrhoeae* clinical isolates with reported ciprofloxacin MICs that were publicly available from the European Nucleotide Archive for strains that would be identified as susceptible by *gyrA* codon 91-based assays.

**Findings:**

Three clinical isolates of *N gonorrhoeae* with substitutions in GyrA position 95 associated with resistance (G or N) maintained intermediate ciprofloxacin MICs (0·125–0·5 μg/mL), which has been associated with treatment failure, despite reversion of GyrA position 91 from phenylalanine to serine. From an in-silico analysis of the 11 355 genomes from *N gonorrhoeae* clinical isolates, we identified 30 isolates with *gyrA* codon 91 encoding a serine and a ciprofloxacin resistance-associated mutation at codon 95. The reported MICs for these isolates varied from 0·023 μg/mL to 0·25 μg/mL, including four with intermediate ciprofloxacin MICs (associated with substantially increased risk of treatment failure). Finally, through experimental evolution, one clinical isolate of *N gonorrhoeae* bearing GyrA 91S acquired ciprofloxacin resistance through mutations in the gene encoding for the B subunit of DNA gyrase (*gyrB*) that also conferred reduced susceptibility to zoliflodacin (ie, MIC ≥2 μg/mL).

**Interpretation:**

Diagnostic escape from *gyrA* codon 91 diagnostics could occur through either reversion of the *gyrA* allele or expansion of circulating lineages. *N gonorrhoeae* genomic surveillance efforts might benefit from including *gyrB*, given its potential for contributing to ciprofloxacin and zoliflodacin resistance, and diagnostic strategies that reduce the likelihood of escape, such as the incorporation of multiple target sites, should be investigated. Diagnostics that guide antibiotic therapy can have unintended consequences, including novel resistance determinants and antibiotic cross-resistance.

**Funding:**

US National Institutes of Health National Institute of Allergy and Infectious Diseases, National Institute of General Medical Sciences, and the Smith Family Foundation.

## Introduction

*Neisseria gonorrhoeae*, the causative bacterium of the sexually transmitted infection gonorrhoea, has developed resistance to each of the first-line antibiotics used to treat it.^1^ This rise in antibiotic resistance has led to fears of untreatable, highly drug-resistant gonorrhoea,^2^ a concern further heightened by the international circulation of strains resistant to the current first-line antibiotic, ceftriaxone.^3^

Due to high levels of drug resistance in *N gonorrhoeae*, there has been substantial interest in the development of new approaches for management of gonorrhoea.^4^ One promising avenue has been the use of point-of-care diagnostics that test for susceptibility. By understanding alleles associated with antibiotic susceptibility,^5^ sequence-based diagnostics might enable diagnosis of gonorrhoea and inform treatment decisions by using genotype to predict antibiotic susceptibility phenotype.

An early target for this approach has been the fluoroquinolone-class antibiotic ciprofloxacin.^6^ Once first-line treatment for *N gonorrhoeae* infections, rising levels of resistance and concerns around failure resulted in recommendations against the empirical use of ciprofloxacin.^7^ However, surveillance data from the USA, the UK, and Europe indicate that ciprofloxacin resistance is present in a few cases,^4^ suggesting that a diagnostic test that can inform on ciprofloxacin susceptibility would allow for the drug’s reintroduction to gonorrhoea treatment and thereby aid in reducing the selective pressure on ceftriaxone.

Ciprofloxacin resistance in *N gonorrhoeae* is mainly mediated by substitutions in the A subunit of DNA gyrase (encoded by *gyrA*) and the A subunit of topoisomerase IV (encoded by *parC*). Although multiple substitutions contribute to ciprofloxacin resistance, including position 95 in GyrA and multiple sites at positions 86–91 in ParC,^8^ position 91 in GyrA is highly predictive of clinical susceptibility (if a serine) and resistance (if a phenylalanine) to ciprofloxacin.^9,10^ A clinical trial in the USA showed confirming by PCR that codon 91 of *gyrA* codes for S, followed by ciprofloxacin treatment, is 100% efficacious at curing gonorrhoea.^11^

The SpeeDx ResistancePlus GC diagnostic test reports the presence of *N gonorrhoeae* and ciprofloxacin susceptibility. The test consists of a multiplex quantitative PCR (qPCR), incorporating channels for the wild-type *gyrA* allele encoding 91S, the mutant *gyrA* allele encoding 91F, the *N gonorrhoeae opa* gene, the *N gonorrhoeae porA* gene, and a PCR positive control. Across several studies, the ResistancePlus assay detected gonorrhoea with a sensitivity of 94–100%^6,12^ and specificity of 99–100%.^6,12^ The test is also highly sensitive (>99%) and specific (>97%) for detection of ciprofloxacin resistance.^13^ The ResistancePlus GC assay has been approved for use in Australia, New Zealand, and the EU.^14^

The use of sequence-based diagnostics raises the possibility of diagnostic escape, with a precedent in *N gonorrhoeae* of mutations that resulted in failure of clinical identification by PCR.^15^ In this case, the diagnostic applies selective pressure for *N gonorrhoeae* to develop resistance through a pathway other than the targeted mutation at codon 91 of *gyrA*. False-negative tests for antibiotic susceptibility testing might result in clinical treatment failure for the individual and increased burden of disease and accelerated spread of resistance in the population.^16^ The risk of ciprofloxacin resistance arising through novel pathways is concerning, as these pathways might enable increased resistance to other antibiotics that, similarly to ciprofloxacin, target bacterial topoisomerases. One such antibiotic is zoliflodacin, a first-in-class oral spiropyrimidinetrione antibiotic that primarily targets the B subunit of DNA gyrase (*gyrB*),^17^ and is in phase 3 trials for treatment of gonorrhoea.^18^

**Figure 1:**
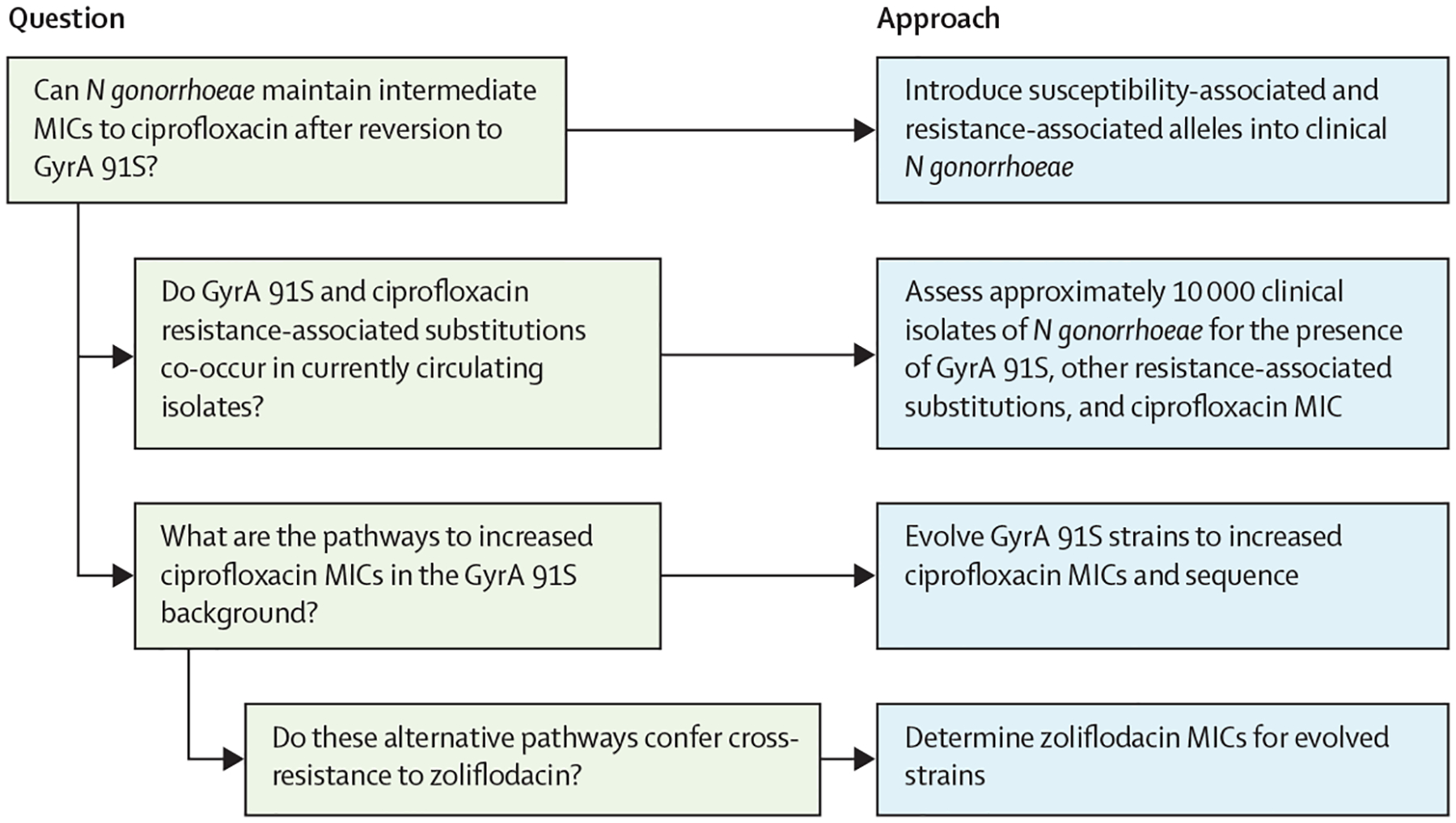
Rationale and methodology for experiments in the study MIC=minimum inhibitory concentration.

In this study, we investigated the pathways of diagnostic escape from tests that rely on *gyrA* codon 91. The goal of this work was to answer three questions that are crucial to understanding ciprofloxacin diagnostic escape in *N gonorrhoeae* (figure 1). First, can *N gonorrhoeae* maintain intermediate minimum inhibitory concentrations (MICs) to ciprofloxacin following reversion from GyrA91F to GyrA 91S? Second, through what GyrA-independent pathways can GyrA 91S isolates acquire increased MICs to ciprofloxacin and are these pathways present in circulating isolates? Third, does the acquisition of increased ciprofloxacin MICs lead to cross-resistance with zoliflodacin, an antibiotic with a similar target?

## Methods

### Bacterial isolates

In this bacterial genetics study, we used strains that are listed in the appendix (pp 3–4). As *N gonorrhoeae* is a biosafety level (BSL)-2 pathogen, no work in this study was performed under BSL-3 conditions. The five isolates for introduction of *gyrA* alleles (table 1) were chosen from a global collection based on two major factors: high-level ciprofloxacin resistance (≥10 μg/mL) and diversity of genetic background. All five isolates encode GyrA S91F as well as an additional substitution in GyrA at position 95 and substitutions in ParC that are known to cause an increased MIC to ciprofloxacin, including ParC S87R/N and ParC E91G/K.^8^ Finally, all five strains had the *gyrB* allele encoding 429D, which is associated with zoliflodacin susceptibility.^19^

### Procedures

*N gonorrhoeae* was grown on gonococcal base (GCB) agar (Becton Dickinson, Franklin Lakes, NJ, USA) supplemented with Kellogg’s supplements (GCB-K; made as 100 × solution in 100 mL, with 40 g glucose [Sigma-Aldrich, St Louis, MO, USA], 0·5 g glutamine [Sigma-Aldrich], 50 mg ferric nitrate (ThermoFisher, Waltham, MA, USA), and 2 mg co-carboxylase [Sigma-Aldrich])^20^ at 37°C in 5% CO_2_. Antibiotic susceptibility testing was performed on GCB-K agar via agar dilution for zoliflodacin (using the range 0·008–4 μg/mL) and Etest (BioMerieux, Marcy-l’Etoile, Auvergene-Rhône-Alpes, France) for ciprofloxacin, ceftriaxone, erythromycin, benzylpenicillin, and tetracycline. All MIC tests were done in duplicate on independent days. Ciprofloxacin MICs were assessed according to the Clinical and Laboratory Standards Institute (CLSI) breakpoints^21^ and isolates were classified as susceptible (MIC ≤0·062 μg/mL), intermediate (MIC 0·125–0·5 μg/mL), or resistant (MIC ≥1 μg/mL). Reduced susceptibility to zoliflodacin is defined as an MIC of 2 μg/mL or more.^19^

For growth curves, *N gonorrhoeae* from an overnight streak was diluted to an optical density at 600 nm (OD_600_) of 0·01 at time zero in phosphate-buffered gonococcal medium (15 g/L proteose peptone 3 [ThermoFisher], 1 g/L soluble starch [ThermoFisher, 4 g/L di-potassium hydrogen phosphate [Sigma-Aldrich], 1 g/L potassium dihydrogen phosphate [Sigma-Aldrich], and 5 g/L sodium chloride [Sigma-Aldrich])^22^ with Kellogg’s supplement with six replicates. At multiple timepoints, OD_600_ was measured with a spectrophotometer and colony-forming units were measured via serial dilution and plating on GCB-K. The experiment was performed independently on two different days.

For *N gonorrhoeae* cloning of *gyrA* and *gyrB* mutants, pairwise combinations of *gyrA* codon 91 alleles (encoding 91S [susceptible] or 91F [resistant]) and codon 95 alleles (95D [susceptible] or 95G and 95N [resistant]) were introduced into these isolates using kanamycin co-selection. To investigate the relationship between *gyrB* and ciprofloxacin resistance, the *gyrB* allele encoding for GyrB D429N was introduced into strain GCGS0481 with GyrA 91S/95G. Primers and plasmids are listed in the appendix (pp 3–4) and methods for cloning are described in the appendix (p 2).

To investigate the relationship between *gyrA* codon 91 and ciprofloxacin MICs, we assessed 11 355 genomes of *N gonorrhoeae* isolates with reported ciprofloxacin MICs. Sequences were accessed via the European Nucleotide Archive and analysed as in a previous study;^5^ methods are described in the appendix (p 2).

We used an experimental evolution approach to assess the possibility that *N gonorrhoeae* isolates with GyrA 91S could acquire higher ciprofloxacin MICs. Strain GCGS0481 with GyrA 91S/95G was grown overnight on GCB-K agar. The overnight streak was scraped, diluted to an OD_600_ of approximately 0·1, then plated as 14 replicates overnight onto GCB-K agar supplemented with 0·125 μg/mL ciprofloxacin. Each replicate was sequentially passaged, following the same dilution pattern, onto double the concentration of ciprofloxacin overnight until there was no visible growth or until 2 μg/mL was reached. Single colonies from the last resulting viable passage were selected for further analysis.

### Statistical analysis

To determine the mutations responsible for increased ciprofloxacin MICs during experimental evolution, the parental isolate GCGS0481 with GyrA 91S/95G and derived isolates were sequenced using the Illumina platform (Illumina, San Diego, CA, USA); the resulting reads are publicly available (see Data Sharing statement at the end of this paper). For reads from the experimentally evolved isolates and publicly available clinical isolates, the genomic analysis pipeline and methods for read mapping are described in the appendix (p 2). To determine the ciprofloxacin MIC of strains with GyrA 91S and a GyrA substitution at position 95 associated with resistance (G, N, or A), we performed an in-silico analysis of the 11 355 genomes from the clinical isolates. This study used data that are publicly available and did not require approval from an institutional review board.

### Role of the funding source

The funders of the study had no role in study design, data collection, data analysis, data interpretation, or writing of the report.

## Results

**Figure 2:**
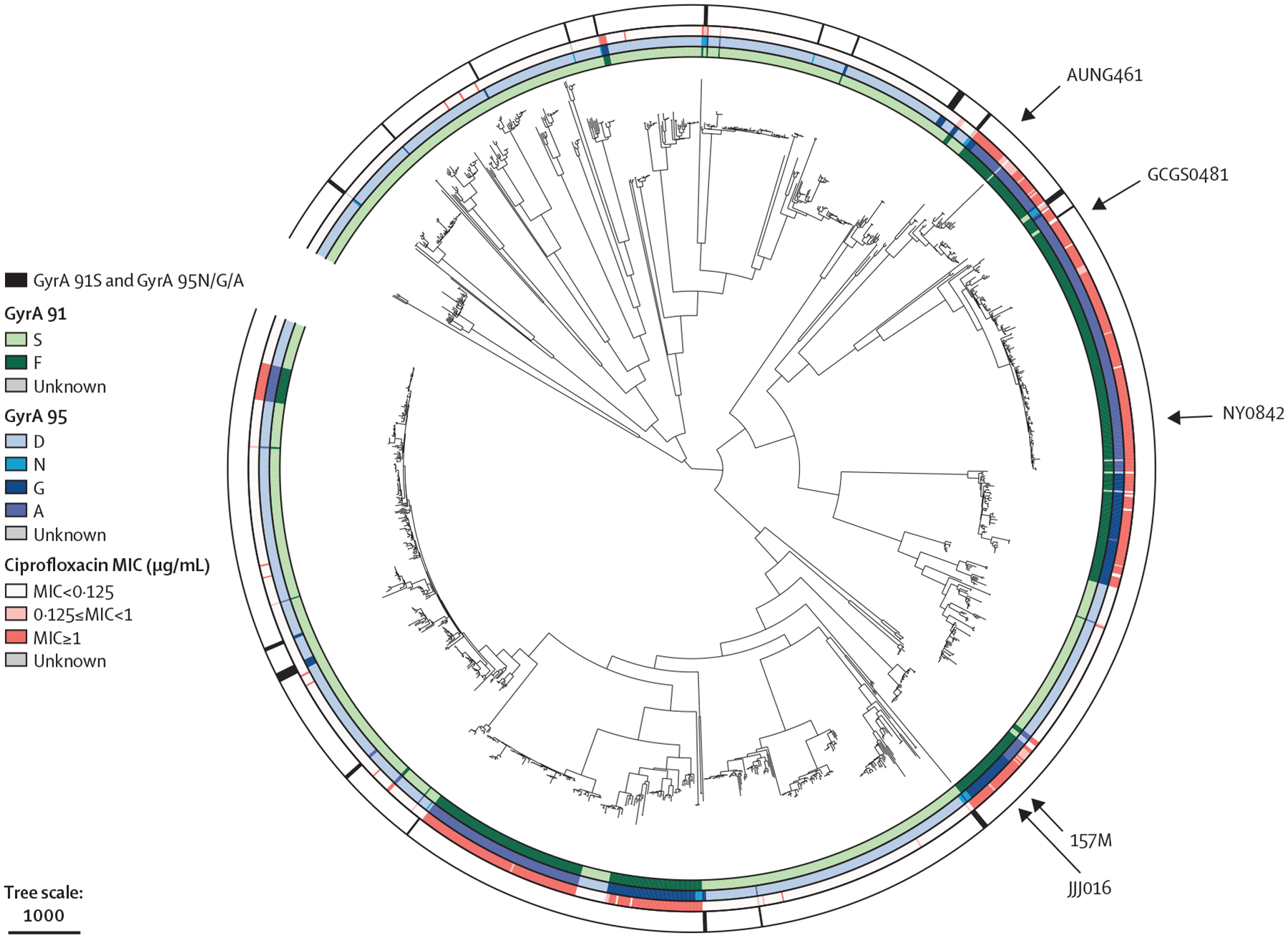
Recombination-adjusted phylogenetic tree of 1510 *Neisseria gonorrhoeae* clinical isolates On the innermost ring, the amino acid at position 91 of GyrA is coded by colour. On the second ring, the amino acid at position 95 of GyrA is also coded by colour. The ciprofloxacin MIC of each isolate is shown on the third ring in red, and isolates with GyrA 91S and GyrA 95N/G/A are shown on the outermost ring in black. Isolates from this study are marked by arrows. Tree scale represents the recombination-adjusted number of single-nucleotide polymorphisms. MIC=minimum inhibitory concentration.

The five strains of *N gonorrhoeae* (figure 2) bearing mutant alleles of *gyrA* had a broad range of MICs. We were particularly interested in strains with GyrA 91S, as these isolates would be reported as ciprofloxacin susceptible by assays focused on this *gyrA* codon 95. There was substantial MIC variation with the susceptible allele at both codons 91 and 95 (encoding GyrA 91S/95D), with MICs varying almost six-fold (table 1).

Introduction of mutations at codon 95, encoding either GyrA 95G or GyrA 95N, increased the MIC in all backgrounds by ten-fold or more. Two isolates—NY0842, a 2013 urethral isolate from New York City (NY, USA), and GCGS0481, a 2006 urethral isolate from the US Department of Health and Human Services region 10 (AK, ID, OR, and WA)—reached an intermediate level of ciprofloxacin MIC with either mutation at the 95 codon; isolate JJJ016 reached an intermediate ciprofloxacin MIC with the GyrA 95N substitution. Notably, MICs for ciprofloxacin of 0·125 μg/mL or more are associated with substantially increased risk of treatment failure.^23^ Finally, introduction of GyrA 91F/95G or 91F/95N into all five backgrounds did not substantially affect MICs compared with the original isolates (table 1).

**Figure 3:**
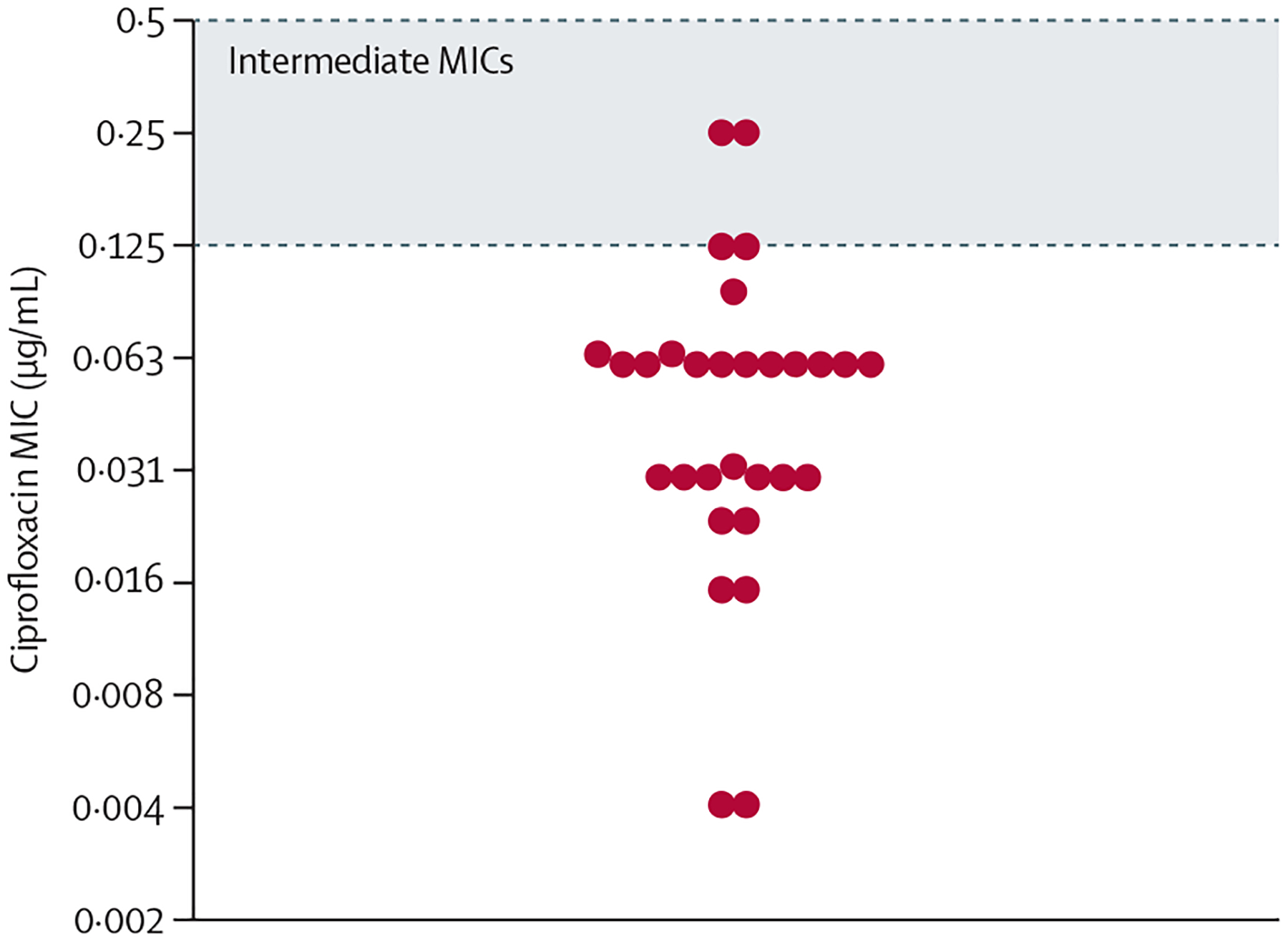
Ciprofloxacin MICs of strains with GyrA 91S and GyrA 95N/G/A shown in figure 2 The Clinical and Laboratory Standards Institute range for intermediate MICs is marked by a red dashed line. MIC=minimum inhibitory concentration.

These data suggest that some clinical isolates still retain ciprofloxacin MICs of 0·125 μg/mL or more in the presence of substitutions in GyrA at position 95, even after the GyrA F91S reversion. The in-silico analysis of 11 355 genomes from clinical isolates identified 30 isolates bearing GyrA 91S/95G, 91S/95N, or 91S/95A, distributed across the *N gonorrhoeae* phylogeny as singletons and closely related isolates (figure 2). The reported MICs for these isolates varied from 0·023 μg/mL to 0·25 μg/mL, including four with intermediate MICs (figure 3).

We investigated the possibility that isolates with GyrA 91S could develop higher-level ciprofloxacin MICs through experimental evolution. We focused on the GyrA 91S/95G mutant of GCGS0481, differing from the parental isolate by only the kanamycin cassette and the 91S allele of *gyrA*, as validated by whole-genome sequencing. Of the 14 parallel cultures, ten (78%) had no viable colonies at 0·25 μg/mL of ciprofloxacin—the MIC before evolution. Single colonies from the four remaining cultures were then re-streaked and tested for ciprofloxacin MICs. One colony had a high ciprofloxacin MIC (>32 μg/mL), attributable to a GyrA S91F substitution; this colony was excluded from further analysis.

All three remaining mutants were isolated at 1 μg/mL ciprofloxacin, suggesting an increased MIC as compared with the GCGS0481 GyrA 91S/95G strain (MIC 0·25 μg/mL; table 2) and clinical resistance to ciprofloxacin by CLSI thresholds (MIC ≥1 μg/mL).^21^ Whole-genome sequencing revealed that all three mutants had maintained the *gyrA* 91S allele and acquired substitutions in *gyrB*; two of the three mutants had no other coding sequence polymorphisms (appendix pp 5–10). One isolate had a missense mutation encoding a GyrB E469D substitution. Introduction of this single-nucleotide polymorphism in the parental isolate resulted in the same ciprofloxacin MIC as the evolved strain and conferred a two-fold increase in the MIC for zoliflodacin (table 2).

Two of the evolved strains gained GyrB D429N substitutions, which have been shown to cause reduced susceptibility to zoliflodacin.^17,19^ Introduction of the mutation encoding the GyrB D429N substitution into the parental isolate recapitulated the phenotype of an increased MIC for ciprofloxacin and led to an eight-fold increase in the MIC for zoliflodacin to 2 μg/mL, as compared with the wildtype and parental GCGS0481 isolates (table 2). These changes in antibiotic susceptibility are specific to ciprofloxacin and zoliflodacin, as the MICs for ceftriaxone, erythromycin, benzylpenicillin, and tetracycline did not vary between the GCGS0481 strain with GyrA 91S/95G and the transformant GCGS0481 strain with GyrA 91S/95G GyrB D429N (appendix p 11). Finally, the *gyrB*-directed transformant, GCGS0481 with GyrA 91S/95G GyrB D429N, grew similarly to the isogenic strain GCGS0481 GyrA 91S/95G (appendix p 12).

## Discussion

In this study, we found that some clinical isolates with a 91S allele of *gyrA* maintained ciprofloxacin MICs at a level that has been associated with treatment failure and that one clinical isolate (GCGS0481) in which we introduced a GyrA F91S substitution acquired ciprofloxacin resistance while maintaining the 91S allele. Although these strains are zoliflodacin-naive, we found that, in the presence of ciprofloxacin, clinical isolates evolved a D429N substitution in GyrB that conferred an increased ciprofloxacin MIC and was associated with zoliflodacin reduced susceptibility.^24^ Taken together, these results indicate that there are evolutionary pathways that could lead to diagnostic escape from *gyrA* codon 91-based diagnostics and that one of these pathways could lead to cross-resistance between ciprofloxacin and zoliflodacin.

As an in-vitro study, this work has limitations for translation to in-vivo clinical *N gonorrhoeae* infections. We used a kanamycin resistance cassette for co-selection and worked with paired isogenic strains that were kanamycin resistant. The kanamycin cassette used in this study is a kanamycin phosphotransferase and therefore acts through drug inactivation—a mechanism that is probably non-overlapping with ciprofloxacin resistance. Because strains expressing the kanamycin cassette had similar MICs to parental isolates with the same gyrase mutations (table 1), we believe that the co-selected kanamycin cassette at most minimally affected the ciprofloxacin MIC profile. These MICs, however, are at the upper range of detection, so we cannot rule out the possibility that the kanamycin cassette has effects on ciprofloxacin MIC or pathways to ciprofloxacin resistance. Furthermore, although the *gyrB* mutants described in this study can be isolated in the laboratory, their in-vivo viability is not known. The method of sequential passaging on ciprofloxacin used to identify these mutations might not replicate the evolutionary pressure applied by antibiotic in the in-vivo setting. Finally, as only 30 clinical isolates of the 11 355 bore GyrA 91S and substitutions in GyrA position 95 associated with non-susceptibility, most of the sequenced isolates at present would probably be correctly identified by *gyrA* codon 91-based diagnostics. This is consistent with the 100% efficacy reported for using ciprofloxacin in the context of infections with *N gonorrhoeae* isolates with GyrA 91S.^11^

Our results indicate that mutations in *gyrB* encoding the D429N substitution can be acquired by *N gonorrhoeae* in the presence of ciprofloxacin and that the GyrB D429N substitution increases ciprofloxacin MICs in at least some backgrounds. As such, this study raises both conceptual and practical implications for the introduction of assays using *gyrA* codon 91 to infer ciprofloxacin susceptibility. From a conceptual standpoint, this study provides an example of how escape from a diagnostic used to guide antibiotic therapy could lead to the acquisition and possible spread of novel resistance determinants and how these pathways might have unintended consequences for resistance profiles to other antibiotics.^16^ The presence of multiple clinical isolates with gyrA 91S, together with resistance-associated substitutions in GyrA position 95, suggest that reversion of codon 91 is viable for pathogenesis. Our findings, although limited to a small subset of genetic backgrounds, also indicate that a test that detects both codons 91 and 95 would be less likely to select for reversion to the susceptible allele, as all of the strains with GyrA 91S/95D in this study remained highly susceptible to ciprofloxacin. A second method for preventing diagnostic escape might be to test for other known ciprofloxacin resistance determinants, including mutations in *parC*. Although the GyrB E469D substitution identified in this study has not been observed in clinical isolates of *N gonorrhoeae*, the corresponding GyrB E466D substitution in clinical isolates of *Salmonella enterica* serovar Typhimurium confers decreased susceptibility to ciprofloxacin.^25^ This finding provides further evidence consistent with the hypothesis that diagnostic escape from a *gyrA* codon 91-based assay might provide evolutionary pressure to drive novel resistance mechanisms. On the whole, these results imply that introduction of a *gyrA* codon 91-based diagnostic to guide therapy—a crucial tool for addressing the challenge of drug resistance in *N gonorrhoeae*—is effective at present,^11^ and its use might be optimally maintained when paired with systematic genomic surveillance to detect escape mutations.

The finding that GyrB D429N reduces susceptibility to both ciprofloxacin and zoliflodacin was unexpected. A previous study of zoliflodacin^24^ showed that, in clinical isolates from China, in-vitro introduction of GyrB D429N led to a decrease in ciprofloxacin susceptibility or had no effect on ciprofloxacin MIC. Other studies have suggested that no cross-resistance between ciprofloxacin and zoliflodacin exists in recently sampled clinical isolates,^26^ probably due to the finding that mutations in *gyrB* encoding the D429N substitution have not been identified in large genomic analyses of *N gonorrhoeae*.^27,28^ One limitation of our study, therefore, is that the GyrB D429N substitution has yet to be identified in clinical isolates. With regard to cross-resistance in vitro, enzymology studies suggest that ciprofloxacin has modestly decreased potency (measured as the concentration at which 50% of the gyrase-DNA cleaved complex is stabilised [CC_50_]) with GyrB D429N compared with GyrB 429D (mean CC_50_ 3·0 [SD 0·4] for the mutant *vs* 1·9 [0·7] for the wild-type).^19^ The cleaved complex is the adduct of enzyme and DNA formed at DNA nicks that are unable to be repaired in the presence of fluoroquinolones. Because these cleaved complexes are thought to be correlated with, if not the mechanism of, fluoroquinolone-mediated cell death,^29^ these data are consistent with zoliflodacin–ciprofloxacin cross-resistance. Cross-resistance between these two antibiotics has not been identified but has been theorised.^30^ Further studies are required to understand the genetic background dependence of phenotypic outcomes following introduction of GyrB D429N. One possibility is that GyrB D429N is epistatic to GyrA S91F with respect to ciprofloxacin resistance and is therefore less likely to arise in ciprofloxacin-resistant backgrounds.

The findings in this study have implications for the introduction of zoliflodacin as a treatment for gonorrhoea, pending a successful phase 3 trial. From the perspective of reduced susceptibility to zoliflodacin, surveillance for the *gyrB* allele encoding for D429N should be prioritised in settings where assays targeting *gyrA* codon 91 inform treatment decisions. From the perspective of ciprofloxacin use, the emergence of zoliflodacin-resistant isolates might also lead to an increase in ciprofloxacin MIC. The allele encoding for GyrB 429N should therefore be considered among the panel of mutations contributing to ciprofloxacin resistance.

## Supplementary Material

1

## Figures and Tables

**Table 1:** Metadata, resistance determinants, and ciprofloxacin MICs of five clinical isolates of *Neisseria gonorrhoeae* following introduction of *gyrA* alleles via kanamycin co-selection

	Accession	Country	Anatomical site	Year	Ciprofloxacin MIC (μg/mL)	Parental GyrA	Parental ParC	Ciprofloxacin MIC (μg/mL)					
								GyrA 91S/95D mutant	GyrA 91S/95G mutant	GyrA 91S/95N mutant	GyrA 91F/95D mutant	GyrA 91F/95G mutant	GyrA 91F/95N mutant
JJJ016	SRR16683798	Hong Kong	Urethra	2015	>32	GyrA 91F/95N	ParC 86D/87R/91E	0·006	0·064	0·19	0·25	>32	>32
NY0842	ERR2631880	USA	Urethra	2013	>32	GyrA 91F/95A	ParC 86D/87N/91K	0·012	0·19	0·25	0·25	>32	>32
AUNG461	SRR8559420	Australia	Urethra	2017	12	GyrA 91F/95G	ParC 86D/87S/91G	0·004	0·064	0·064	0·19	12	12
GCGS0481	ERR855135	USA	Urethra	2006	>32	GyrA 91F/95G	ParC 86D/87R/91E	0·023	0·25	0·25	0·50	>32	>32
157M	SRR16683705	Viet Nam	Urethra	2019	>32	GyrA 91F/95N	ParC 86D/87R/91E	0·004	0·047	0·094	0·25	>32	>32

MIC=minimum inhibitory concentration.

**Table 2:** Ciprofloxacin and zoliflodacin MICs of *Neisseria gonorrhoeae* isolates with GyrA 91S/95G following experimental evolution in the presence of ciprofloxacin

	Ciprofloxacin MIC (μg/mL)	Zoliflodacin MIC (μg/mL)	Ciprofloxacin concentration at isolation
GCGS0481 GyrA 91F/95G (parent)	>32	0·25	NA
GCGS0481 GyrA 91S/95G	0·25	0·25	NA
Experimental evolution GCGS0481 GyrA 91S/95G, isolate 1 (GyrB E469D)	1·5	0·50	1 μg/mL
GCGS0481 GyrA 91S/95G GyrB E469D	1·5	0·50	NA
Experimental evolution GCGS0481 GyrA 91S/95G, isolate 2 (GyrB D429N)	1·5	2	1 μg/mL
Experimental evolution GCGS0481 GyrA 91S/95G, isolate 3 (GyrB D429N)	1·5	2	1 μg/mL
GCGS0481 GyrA 91S/95G GyrB D429N	1·5	2	NA

MIC=minimum inhibitory concentration. NA=not applicable.

## Research in context

### Evidence before this study

The rising incidence of gonorrhoea and its increasing antibiotic resistance has prompted control efforts that include new diagnostics and therapeutics. One diagnostic strategy predicts resistance to quinolones from the *Neisseria gonorrhoeae* genetic sequence, where *gyrA* codon 91 is highly predictive of susceptibility if coding for a serine, and resistance if coding for a phenylalanine. New therapeutics include zoliflodacin, a spiropyrimidinetrione-class antibiotic that inhibits type II topoisomerase, that is currently in phase 3 trials for treatment of gonorrhoea. We searched PubMed twice for articles published between database inception and Aug 22, 2022, in any language using the search terms (1) either “diagnostic escape” or “false-negative” and “*Neisseria gonorrhoeae*” and (2) “zoliflodacin” and “*Neisseria gonorrhoeae*.” Studies on diagnostic escape related primarily to strains escaping nucleic acid amplification tests used to diagnose *N gonorrhoeae* infection and were not focused on resistance or susceptibility determinants. We did not find reports of cross-resistance between ciprofloxacin and zoliflodacin in *N gonorrhoeae*.

### Added value of this study

To investigate the possibility that use of *gyrA* codon 91-based diagnostics would apply a selective pressure driving diagnostic escape, we introduced combinations of resistance-associated and susceptibility-associated *gyrA* codons 91 and 95 into five clinical isolates of *N gonorrhoeae*, including reverting GyrA 91 from F to S. We found that a subset of these reverted isolates with GyrA 91S had intermediate minimum inhibitory concentrations (MICs) to ciprofloxacin, indicating a heightened risk of treatment failure. We identified clinical isolates from across the *N gonorrhoeae* phylogeny with similar *gyrA* alleles and ciprofloxacin MICs, suggesting that strains at risk of diagnostic and treatment failure are circulating. We found that a clinical isolate that we converted from bearing GyrA 91F to GyrA 91S acquired ciprofloxacin resistance through experimental evolution while maintaining GyrA 91S; the resistance-conferring substitution was in GyrB, which also conferred reduced susceptibility to zoliflodacin.

### Implications of all the available evidence

Gonorrhoea treatment strategies that use ciprofloxacin when *gyrA* codon 91-based diagnostics predict ciprofloxacin susceptibility apply a selective pressure that could drive diagnostic escape and new pathways to ciprofloxacin resistance involving mutations in *gyrB*. Furthermore, these novel resistance pathways might overlap with mechanisms of resistance to zoliflodacin. Reciprocally, use of zoliflodacin might drive *gyrB* mutations that facilitate diagnostic escape from *gyrA* codon 91-based diagnostics for ciprofloxacin susceptibility. These findings define *N gonorrhoeae* mutations that should be monitored via surveillance systems, particularly in populations and regions deploying *gyrA* codon 91-based diagnostics, and demonstrate how diagnostics that guide antibiotic therapy can have unintended consequences.

## Footnotes

Data sharing All whole-genome sequencing data produced in this work are available in SRA BioProject PRJNA901210. The code used in the genomics pipeline is publicly available as described in the appendix (p 2). The GCGS0481 GyrA 91S/95G assembly used for the mapping data in the appendix (pp 5–10) is available on figshare. The remaining data are publicly available or are available from the authors upon request. Strains created in this study are also available from the authors upon request.
