# Supplementary material for: Neisseria gonorrhoeae diagnostic escape from a gyrA-based test for ciprofloxacin susceptibility and the effect on zoliflodacin resistance: a bacterial genetics and experimental evolution study
Source: Lancet Microbe. Author manuscript; Available in PMC 2023 Apr 4. (PMC10071290; doi:10.1016/S2666-5247(22)00356-1)
Supplement: 1 [file NIHMS1887317-supplement-1.pdf]

THE LANCET
Microbe

Supplementary appendix

This appendix formed part of the original submission and has been peer reviewed. We post it as supplied by the authors.

Supplement to: Rubin DHF, Mortimer TD, Grad YH. *Neisseria gonorrhoeae* diagnostic escape from a *gyrA*-based test for ciprofloxacin susceptibility and the effect on zoliflodacin resistance: a bacterial genetics and experimental evolution study. *Lancet Microbe* 2023; published online Feb 28. https://doi.org/10.1016/S2666-5247(22)00356-1.

Appendix.

Table of contents

Supplementary methods – page 2
Cloning – page 2
Genomic Pipeline – page 2
Supplementary Table 1. Strain Table – page 3
Supplementary Table 2. Mapped SNPs in evolved and transformed strains – page 5
Supplementary Table 3. Clinically-relevant MICs in isogenic GyrB^{D429} strains – page 11
Supplementary Figure 1. Growth of isogenic GyrB^{D429N} strains – page 12
References – page 13

Supplementary Methods

Cloning

Cloning was performed using primers and plasmids listed in Supp. Table 1 and Gibson assembly[1] into a pUC19[2] backbone using a kanamycin cassette from pDR1[3]. Fragments were amplified using Phusion (NEB, Ipswich, MA, USA), checked for appropriate size by gel electrophoresis, column purified (Qiagen PCR Purification kit), assembled with Gibson Master Mix (NEB, Ipswich, MA, USA), and finally transformed into chemically competent DH5α *E. coli*. Finally, individual colonies were selected on LB agar with 50 µg/mL kanamycin, picked, and grown overnight. Plasmids were isolated using spin columns (Qiagen Spin Miniprep Kit, Hilden, North Rhine-Westphalia, Germany). The resulting plasmids were checked by Sanger sequencing. For insertion of GyrA alleles into *N. gonorrhoeae*, strains of *N. gonorrhoeae* in Table 1 were grown overnight on GCB-K. After 16-20 hours, strains were scraped into 0.3M sucrose, electroporated (V=1.8 kV, exponentially decaying wave) with 200 ng of plasmids DRE77-82, and rescued with GCP medium with Kellogg's supplement. After 10 minutes of rescue, transformants were then plated on nonselective GCB-K agar for 4 hours followed by selection on GCB-K supplemented with 70 µg/mL kanamycin. Finally, single colonies were re-streaked on GCB-K agar and checked for GyrA allele by Sanger sequencing. For GyrB cloning, fragments of GyrB were amplified using primers DR542 and DR543. Electroporation was performed as above, and single colonies were selected on GCB-K agar supplemented with 0.5 µg/mL ciprofloxacin.

Genomic Pipeline

The genomic pipeline was conducted with methodology as published.[4] Reads and metadata for the sequenced *N. gonorrhoeae* isolates were accessed from multiple sources[5-34]. Reads were then inspected using FastQC (v0.11.7) (https://www.bioinformatics.babraham.ac.uk/projects/fastqc/) and removed based on divergent GC content (from ~52-54%) or poor base quality. The remaining reads were mapped to the genome of *N. gonorrhoeae* strain NCCP11945 (RefSeq accession NC_011035.1) with BWA-MEM (v0.7.17-r1188)[35]. Mapped reads were then deduplicated with Picard (v2.20.1) (https://github.com/broadinstitute/picard). Samples with <40% coverage or <70% reads aligned were discovered with BamQC in Qualimap (v2.2.1)[36] and discarded. Variants, including those in *gyrA* codons 91 and 95, were called with Pilon (v1.23)[37] with a minimum mapping quality of 20 and a minimum depth of 10. Pseudogenomes were generated from the resulting Pilon VCFs with inclusion of all PASS sites and alternate alleles with allele frequency greater than 0.9. All other sites were set to "N", and finally samples with >15% of sites called as missing were excluded. SPAdes (v3.12.0, with 8 threads, the –careful flag, and paired end reads where available)[38] was used to create *de novo* assemblies, followed by contig filtering for quality (coverage >10X, length >500 bp, total genome size ~2.0-2.4 Mbp). These assemblies were annotated using Prokka (v1.14.6)[39]. Finally, the phylogenetic tree in this study was assembled in a recombination-corrected manner using Gubbins (v2.3.4)[40] from all sequenced isolates from Reimche *et al.*[27], representing lineages recently transmitting in the US, as well as the five genetically manipulated strains in Table 1 and all strains that had $GyrA^{91S}$ and $GyrA^{95G/A/N}$. The phylogeny was visualized in iTOL (v6)[41].

Transformants in this study were sequenced at SeqCenter (seqcenter.com). Sample libraries were assembled using the Illumina DNA Prep Kit and IDT 10bp UDI indices. The resulting libraries were sequenced with an Illumina NextSeq 2000. The paired-end 2x151 bp reads were demultiplexed, followed by quality control and adapter trimming, using bcl-convert (v3.9.3) (https://support.illumina.com/sequencing/sequencing_software/bcl-convert.html). Resulting reads were merged using BBMerge (v38.97)[42]. Reads were mapped to the parental genomic DNA assembly and variants predicted in Geneious 2021.0 (www.geneious.com) with a minimum coverage of 4 and a variant frequency of >80%.

Plasmid/Strain	Property(ies)	Citation
**Plasmids**		
DRE77	pUC19 with AphA3 Kan^{R} cassette + homology to clone GyrA$^{91S/95D}$	This Study
DRE78	pUC19 with AphA3 Kan^{R} cassette + homology to clone GyrA$^{91F/95D}$	This Study
DRE79	pUC19 with AphA3 Kan^{R} cassette + homology to clone GyrA$^{91S/95N}$	This Study
DRE80	pUC19 with AphA3 Kan^{R} cassette + homology to clone GyrA$^{91F/95G}$	This Study
DRE81	pUC19 with AphA3 Kan^{R} cassette + homology to clone GyrA$^{91S/95N}$	This Study
DRE82	pUC19 with AphA3 Kan^{R} cassette + homology to clone GyrA$^{91F/95N}$	This Study
**Strains**		
JJJ016_SD	Clinical *N. gonorrhoeae* isolate JJJ016, Kan^{R}, GyrA$^{91S/95D}$	This Study
JJJ016_FD	Clinical *N. gonorrhoeae* isolate JJJ016, Kan^{R}, GyrA$^{91F/95D}$	This Study
JJJ016_SN	Clinical *N. gonorrhoeae* isolate JJJ016, Kan^{R}, GyrA$^{91S/95N}$	This Study
JJJ016_FG	Clinical *N. gonorrhoeae* isolate JJJ016, Kan^{R}, GyrA$^{91F/95G}$	This Study
JJJ016_SN	Clinical *N. gonorrhoeae* isolate JJJ016, Kan^{R}, GyrA$^{91S/95N}$	This Study
JJJ016_FN	Clinical *N. gonorrhoeae* isolate JJJ016, Kan^{R}, GyrA$^{91F/95N}$	This Study
NY0842_SD	Clinical *N. gonorrhoeae* isolate NY0842, Kan^{R}, GyrA$^{91S/95D}$	This Study
NY0842_FD	Clinical *N. gonorrhoeae* isolate NY0842, Kan^{R}, GyrA$^{91F/95D}$	This Study
NY0842_SN	Clinical *N. gonorrhoeae* isolate NY0842, Kan^{R}, GyrA$^{91S/95N}$	This Study
NY0842_FG	Clinical *N. gonorrhoeae* isolate NY0842, Kan^{R}, GyrA$^{91F/95G}$	This Study
NY0842_SN	Clinical *N. gonorrhoeae* isolate NY0842, Kan^{R}, GyrA$^{91S/95N}$	This Study
NY0842_FN	Clinical *N. gonorrhoeae* isolate NY0842, Kan^{R}, GyrA$^{91F/95N}$	This Study
AUNG461_SD	Clinical *N. gonorrhoeae* isolate AUNG461, Kan^{R}, GyrA$^{91S/95D}$	This Study
AUNG461_FD	Clinical *N. gonorrhoeae* isolate AUNG461, Kan^{R}, GyrA$^{91F/95D}$	This Study
AUNG461_SN	Clinical *N. gonorrhoeae* isolate AUNG461, Kan^{R}, GyrA$^{91S/95N}$	This Study
AUNG461_FG	Clinical *N. gonorrhoeae* isolate AUNG461, Kan^{R}, GyrA$^{91F/95G}$	This Study
AUNG461_SN	Clinical *N. gonorrhoeae* isolate AUNG461, Kan^{R}, GyrA$^{91S/95N}$	This Study
AUNG461_FN	Clinical *N. gonorrhoeae* isolate AUNG461, Kan^{R}, GyrA$^{91F/95N}$	This Study
GCGS0481_SD	Clinical *N. gonorrhoeae* isolate GCGS0481, Kan^{R}, GyrA$^{91S/95D}$	This Study
GCGS0481_FD	Clinical *N. gonorrhoeae* isolate GCGS0481, Kan^{R}, GyrA$^{91F/95D}$	This Study
GCGS0481_SN	Clinical *N. gonorrhoeae* isolate GCGS0481, Kan^{R}, GyrA$^{91S/95N}$	This Study
GCGS0481_FG	Clinical *N. gonorrhoeae* isolate GCGS0481, Kan^{R}, GyrA$^{91F/95G}$	This Study
GCGS0481_SN	Clinical *N. gonorrhoeae* isolate GCGS0481, Kan^{R}, GyrA$^{91S/95N}$	This Study
GCGS0481_FN	Clinical *N. gonorrhoeae* isolate GCGS0481, Kan^{R}, GyrA$^{91F/95N}$	This Study
157M_SD	Clinical *N. gonorrhoeae* isolate 157M, Kan^{R}, GyrA$^{91S/95D}$	This Study
157M_FD	Clinical *N. gonorrhoeae* isolate 157M, Kan^{R}, GyrA$^{91F/95D}$	This Study
157M_SN	Clinical *N. gonorrhoeae* isolate 157M, Kan^{R}, GyrA$^{91S/95N}$	This Study
157M_FG	Clinical *N. gonorrhoeae* isolate 157M, Kan^{R}, GyrA$^{91F/95G}$	This Study
157M_SN	Clinical *N. gonorrhoeae* isolate 157M, Kan^{R}, GyrA$^{91S/95N}$	This Study
157M_FN	Clinical *N. gonorrhoeae* isolate 157M, Kan^{R}, GyrA$^{91F/95N}$	This Study

Strains cont.		
Experimental evolution GCGS0481_SG, isolate 1 (GyrB^{E469D})	GCGS0481_SG, Kan^{R}, isolated following sequential passage from ciprofloxacin 1 µg/mL passage, encodes GyrB^{E469D}	This Study
GCGS0481_SG GyrB^{E469D}	GCGS0481_SG, Kan^{R}, GyrB^{E469D}	This Study
Experimental evolution GCGS0481_SG, isolate 2 (GyrB^{D429N})	GCGS0481_SG, Kan^{R}, isolated following sequential passage from ciprofloxacin 1 µg/mL passage, encodes GyrB^{D429N}	This Study
Experimental evolution GCGS0481_SG, isolate 3 (GyrB^{D429N})	GCGS0481_SG, Kan^{R}, isolated following sequential passage from ciprofloxacin 1 µg/mL passage, encodes GyrB^{D429N}	This Study
GCGS0481_SG GyrB^{D429N}	GCGS0481_SG, Kan^{R}, GyrB^{D429N}	This Study

Primers	Sequence (Annealing)	Template
DR_487_GyrAGib_1_F	TCCGTAGGTGAACCTGCGGGCGGCTGCTCGGG	NG gDNA
DR_477_GyrAGib_1_R	CGGCGGATCCCGCAGACCTTGTCAAAGCCGA	NG gDNA
DR_478_GyrAGib_2_F	CGGCTTTGACAAGGTCTGCGGGATCCGCCGTC	AphA3 Kan^{R} cassette
DR_479_GyrAGib_2_R	AACATGATTTAAATAACGCGTCGACGCTTTTTA	AphA3 Kan^{R} cassette
DR_480_GyrAGib_3_F	CGACGCGTTATTTAAATCATGTTGCGGGAAAGC	NG gDNA
DR_496_GyrAGib_3_R	GCTAGTTATTGCTCAGCGGGCGGCCTGTTTTATAGCCT	NG gDNA
DR_194_puc19_Gib_F	CCGCTGAGCAATAACTAGCGGATCCCCGGGTACCG	pUC19
DR_195_puc19_Gib_R	CCGCAGGTTCACCTACGGATCTAGAGTCGACCTGCAGG	pUC19
DR_542_gyrB_F	CAAGCAAACCGGAAAGTTCG	NG gDNA
DR_543_gyrB_R	GAAACCGCCGGCGAC	NG gDNA

Supplementary Table 1. Strain Table. Strains, plasmids, and primers used in this study.

Contig	Position	Change	Coverage	Polymorphism Type	Variant Frequency	Amino Acid Change	CDS Position	Codon Change	product	Protein Effect
Experimental evolution GCGS0481 GyrA$^{91S/95G}$, isolate 1										
6	25856	G -> A	364	SNP (transition)	98.40%		328		Type IV pilus biogenesis and competence protein PilQ	Truncation
1	79339	=-T	333	Deletion	100.00%		254		putative protein YqeY	Frame Shift
5	95530	C -> T	1461	SNP (transition)	82.70%		525	GCG -> GCA	hypothetical protein	None
3	85223	G -> T	380	SNP (transversion)	99.20%	E -> D	1407	GAG -> GAT	DNA gyrase subunit B	Substitution
70	1520	C -> G	39	SNP (transversion)	82.10%					
70	1540	A -> G	6	SNP (transition)	83.30%					
70	1543	C -> G	5	SNP (transversion)	100.00%					
107	67	A -> T	8	SNP (transversion)	100.00%					
107	200	C -> A	8	SNP (transversion)	100.00%					
107	254	C -> A	8	SNP (transversion)	100.00%					
119	70	T -> C	8	SNP (transition)	100.00%					
123	73	T -> A	71	SNP (transversion)	98.60%					
123	124	T -> A	55	SNP (transversion)	100.00%					
123	169	A -> T	45	SNP (transversion)	100.00%					
132	35	T -> A	16	SNP (transversion)	100.00%					
132	68	C -> G	14	SNP (transversion)	100.00%					

224	1	A -> G	5	SNP (transition)	100.00%					
234	78	T -> C	22	SNP (transition)	95.50%					
245	50	T -> A	4	SNP (transversion)	100.00%					
246	49	T -> C	15	SNP (transition)	86.70%					
279	28	A -> T	46	SNP (transversion)	100.00%					
280	78	T -> A	160	SNP (transversion)	93.80%					
291	1	A -> G	16	SNP (transition)	81.30%					
291	3	ACC -> CAG	16	Substitution	81.30%					
292	48	C -> T	19	SNP (transition)	84.20%					
312	82	G -> A	11	SNP (transition)	81.80%					
330	79	T -> C	5	SNP (transition)	80.00%					
332	4	T -> G	8	SNP (transversion)	100.00%					
346	70	A -> C	15	SNP (transversion)	80.00%					
346	75	GA -> TC	13	Substitution	100.00%					
346	78	A -> T	13	SNP (transversion)	92.30%					
Experimental evolution GCGS0481 GyrA$^{91S/95G}$, isolate 2										
3	85101	G -> A	224	SNP (transition)	100.00%	D -> N	1285	GAC -> AAC	DNA gyrase subunit B	Substitution
70	1520	C -> G	31	SNP (transversion)	93.50%					
70	1556	C -> G	22	SNP (transversion)	100.00%					
70	1568	A -> T	23	SNP (transversion)	100.00%					
70	1571	A -> C	23	SNP (transversion)	100.00%					

70	1584	A -> C	25	SNP (transversion)	100.00%					
70	1595	A -> C	28	SNP (transversion)	100.00%					
70	1598	C -> G	28	SNP (transversion)	100.00%					
70	1607	GG -> CT	28	Substitution	100.00%					
70	1613	TG -> GC	30	Substitution	100.00%					
70	1619	A -> T	32	SNP (transversion)	93.80%					
70	1621	A -> T	34	SNP (transversion)	100.00%					
70	1625	T -> G	34	SNP (transversion)	100.00%					
70	1632	A -> C	32	SNP (transversion)	100.00%					
70	1637	C -> A	32	SNP (transversion)	90.60%					
123	73	T -> A	5	SNP (transversion)	100.00%					
186	33	A -> C	170	SNP (transversion)	94.10%					
226	68	A -> G	31	SNP (transition)	100.00%					
226	129	C -> T	16	SNP (transition)	93.80%					
243	124	A -> C	100	SNP (transversion)	82.00%					
248	47	G -> A	10	SNP (transition)	100.00%					
301	19	=+TG	27 -> 29	Insertion	81.5% -> 82.8%					
301	21	T -> C	29	SNP (transition)	86.20%					
310	45	AA -> GG	5	Substitution	100.00%					
310	53	G -> A	5	SNP (transition)	100.00%					
310	55	A -> C	5	SNP (transversion)	100.00%					

Experimental evolution GCGS0481 GyrA$^{91S/95G}$, isolate 3										
3	85101	G -> A	242	SNP (transition)	100.00%	D -> N	1285	GAC -> AAC	DNA gyrase subunit B	Substitution
1	121055	(C)10 -> (C)11	80	Insertion (tandem repeat)	88.80%					
70	1520	C -> G	20	SNP (transversion)	95.00%					
70	1540	A -> G	6	SNP (transition)	83.30%					
104	4	=+G	65	Insertion	87.70%					
123	73	T -> A	17	SNP (transversion)	100.00%					
123	124	T -> A	17	SNP (transversion)	100.00%					
226	68	A -> G	44	SNP (transition)	100.00%					
246	1	TC -> GA	63	Substitution	98.40%					
246	49	T -> C	67	SNP (transition)	98.50%					
252	59	C -> G	11	SNP (transversion)	81.80%					
279	62	-C	5	Deletion	80.00%					
279	105	G -> T	7	SNP (transversion)	85.70%					
295	78	GC -> TT	110	Substitution	99.10%					
301	2	C -> G	4	SNP (transversion)	100.00%					
305	78	T -> C	10	SNP (transition)	80.00%					
305	87	AT -> GC	8	Substitution	87.50%					
GCGS0481 GyrA$^{91S/95G}$ GyrB^{D429N}										
5	95530	C -> T	1610	SNP (transition)	90.40%		525	GCG -> GCA	hypothetical protein	None
3	85101	G -> A	426	SNP (transition)	100.00%	D -> N	1285	GAC -> AAC	DNA gyrase subunit B	Substitution
70	1520	C -> G	67	SNP (transversion)	95.50%					

70	1568	A -> T	23	SNP (transversion)	95.70%					
70	1571	A -> C	22	SNP (transversion)	100.00%					
70	1584	A -> C	22	SNP (transversion)	100.00%					
70	1595	A -> C	21	SNP (transversion)	95.20%					
70	1598	C -> G	19	SNP (transversion)	100.00%					
70	1607	GG -> CT	17	Substitution	100.00%					
70	1613	TG -> GC	17	Substitution	100.00%					
70	1619	A -> T	17	SNP (transversion)	100.00%					
70	1621	A -> T	17	SNP (transversion)	100.00%					
70	1625	T -> G	17	SNP (transversion)	100.00%					
70	1632	A -> C	16	SNP (transversion)	100.00%					
70	1637	C -> A	16	SNP (transversion)	100.00%					
104	4	=+G	97	Insertion	91.80%					
123	73	T -> A	33	SNP (transversion)	100.00%					
123	124	T -> A	42	SNP (transversion)	100.00%					
123	169	A -> T	34	SNP (transversion)	100.00%					
123	35	T -> A	19	SNP (transversion)	100.00%					
132	68	C -> G	21	SNP (transversion)	100.00%					
252	59	C -> G	30	SNP (transversion)	93.30%					
252	120	T -> C	16	SNP (transition)	93.80%					
279	28	A -> T	20	SNP (transversion)	100.00%					

289	73	C -> A	29	SNP (transversion)	93.10%					
305	87	AT -> GC	10	Substitution	90.00%					
338	13	G -> A	8	SNP (transition)	87.50%					
338	15	T -> C	8	SNP (transition)	87.50%					

Supplementary Table 2. Mapped SNPs in evolved and transformed strains. SNPs annotated in experimentally evolved and directly transformed derivatives of strain GCGS0481 GyrA$^{91S/95G}$. The assembly used for mapping is available at https://figshare.com/articles/dataset/GCGS0481_GyrA91S_95G/21554451.

Antibiotic MIC	GCGS0481 GyrA$^{91S/95G}$	GCGS0481 GyrA$^{91S/95G}$ GyrB^{D429N}
Ceftriaxone	0.008 µg/mL	0.008 µg/mL
Erythromycin	4 µg/mL	4 µg/mL
Benzylpenicillin	0.75 µg/mL	0.75 µg/mL
Tetracycline	1 µg/mL	1 µg/mL

Supplementary Table 3. **Clinically-relevant MICs in isogenic GyrB^{D429} strains.** MICs for GCGS0481 GyrA$^{91S/95G}$ and GCGS0481 GyrA$^{91S/95G}$ GyrB^{D429N}.

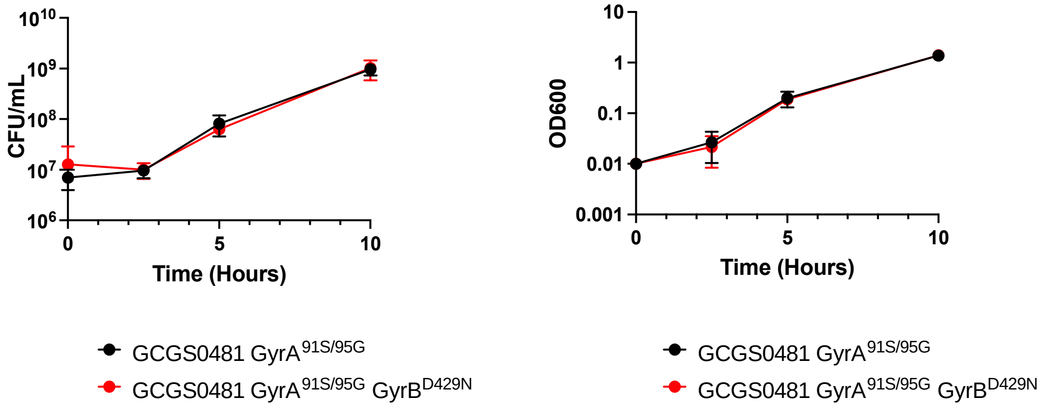

Supplementary Figure 1. **Growth of isogenic GyrB^{D429N} strains.** Growth curves of GCGS0481 GyrA$^{91S/95G}$ and GCGS0481 GyrA$^{91S/95G}$ GyrB^{D429N} in GCP liquid medium with Kellogg's supplements by colony-forming units (CFUs) (Left) and optical density 600 (OD600) (Right). Dots indicate arithmetic mean and error bars represent standard of deviation (N=6). These data are representative of two independent experiments.

References

1. Gibson DG, Young L, Chuang RY, Venter JC, Hutchison CA, 3rd, Smith HO. Enzymatic assembly of DNA molecules up to several hundred kilobases. *Nat Methods* 2009; **6**(5): 343-5.
2. Norrander J, Kempe T, Messing J. Construction of improved M13 vectors using oligodeoxynucleotide-directed mutagenesis. *Gene* 1983; **26**(1): 101-6.
3. Rubin DHF, Ma KC, Westervelt KA, Hullahalli K, Waldor MK, Grad YH. Variation in supplemental carbon dioxide requirements defines lineage-specific antibiotic resistance acquisition in Neisseria gonorrhoeae. *bioRxiv* 2022: 2022.02.24.481660.
4. Ma KC, Mortimer TD, Hicks AL, et al. Increased antibiotic susceptibility in Neisseria gonorrhoeae through adaptation to the cervical environment. *bioRxiv* 2020: 2020.01.07.896696.
5. Alfsnes K, Eldholm V, Olsen AO, et al. Genomic epidemiology and population structure of Neisseria gonorrhoeae in Norway, 2016-2017. *Microb Genom* 2020; **6**(4).
6. Buckley C, Forde BM, Trembizki E, Lahra MM, Beatson SA, Whiley DM. Use of whole genome sequencing to investigate an increase in Neisseria gonorrhoeae infection among women in urban areas of Australia. *Scientific reports* 2018; **8**(1): 1503.
7. Cehovin A, Harrison OB, Lewis SB, et al. Identification of Novel Neisseria gonorrhoeae Lineages Harboring Resistance Plasmids in Coastal Kenya. *J Infect Dis* 2018; **218**(5): 801-8.
8. Demczuk W, Lynch T, Martin I, et al. Whole-genome phylogenomic heterogeneity of Neisseria gonorrhoeae isolates with decreased cephalosporin susceptibility collected in Canada between 1989 and 2013. *J Clin Microbiol* 2015; **53**(1): 191-200.
9. Demczuk W, Martin I, Peterson S, et al. Genomic Epidemiology and Molecular Resistance Mechanisms of Azithromycin-Resistant Neisseria gonorrhoeae in Canada from 1997 to 2014. *J Clin Microbiol* 2016; **54**(5): 1304-13.
10. De Silva D, Peters J, Cole K, et al. Whole-genome sequencing to determine transmission of Neisseria gonorrhoeae: an observational study. *The Lancet Infectious diseases* 2016; **16**(11): 1295-303.
11. Didelot X, Dordel J, Whittles LK, et al. Genomic Analysis and Comparison of Two Gonorrhea Outbreaks. *mBio* 2016; **7**(3).
12. Eyre DW, De Silva D, Cole K, et al. WGS to predict antibiotic MICs for Neisseria gonorrhoeae. *The Journal of antimicrobial chemotherapy* 2017; **72**(7): 1937-47.
13. Ezewudo MN, Joseph SJ, Castillo-Ramirez S, et al. Population structure of Neisseria gonorrhoeae based on whole genome data and its relationship with antibiotic resistance. *PeerJ* 2015; **3**: e806.
14. Fifer H, Cole M, Hughes G, et al. Sustained transmission of high-level azithromycin-resistant Neisseria gonorrhoeae in England: an observational study. *The Lancet Infectious diseases* 2018; **18**(5): 573-81.
15. Gernert KM, Seby S, Schmerer MW, et al. Azithromycin susceptibility of Neisseria gonorrhoeae in the USA in 2017: a genomic analysis of surveillance data. *Lancet Microbe* 2020; **1**(4): e154-e64.
16. Golparian D, Bazzo ML, Golfetto L, et al. Genomic epidemiology of Neisseria gonorrhoeae elucidating the gonococcal antimicrobial resistance and lineages/sublineages across Brazil, 2015-16. *The Journal of antimicrobial chemotherapy* 2020; **75**(11): 3163-72.
17. Grad YH, Harris SR, Kirkcaldy RD, et al. Genomic Epidemiology of Gonococcal Resistance to Extended-Spectrum Cephalosporins, Macrolides, and Fluoroquinolones in the United States, 2000-2013. *J Infect Dis* 2016; **214**(10): 1579-87.
18. Harris SR, Cole MJ, Spiteri G, et al. Public health surveillance of multidrug-resistant clones of Neisseria gonorrhoeae in Europe: a genomic survey. *The Lancet Infectious diseases* 2018; **18**(7): 758-68.

19. de Korne-Elenbaas J, Bruisten SM, de Vries HJC, Van Dam AP. Emergence of a Neisseria gonorrhoeae clone with reduced cephalosporin susceptibility between 2014 and 2019 in Amsterdam, The Netherlands, revealed by genomic population analysis. *J Antimicrob Chemother* 2021; **76**(7): 1759-68.
20. Kwong JC, Chow EPF, Stevens K, et al. Whole-genome sequencing reveals transmission of gonococcal antibiotic resistance among men who have sex with men: an observational study. *Sexually transmitted infections* 2018; **94**(2): 151-7.
21. Lan PT, Golparian D, Ringlander J, Van Hung L, Van Thuong N, Unemo M. Genomic analysis and antimicrobial resistance of Neisseria gonorrhoeae isolates from Vietnam in 2011 and 2015-16. *The Journal of antimicrobial chemotherapy* 2020; **75**(6): 1432-8.
22. Lee RS, Seemann T, Heffernan H, et al. Genomic epidemiology and antimicrobial resistance of Neisseria gonorrhoeae in New Zealand. *The Journal of antimicrobial chemotherapy* 2018; **73**(2): 353-64.
23. Mortimer TD, Pathela P, Crawley A, et al. The Distribution and Spread of Susceptible and Resistant Neisseria gonorrhoeae Across Demographic Groups in a Major Metropolitan Center. *Clinical infectious diseases : an official publication of the Infectious Diseases Society of America* 2021; **73**(9): e3146-e55.
24. Parmar NR, Singh R, Martin I, et al. Genomic Analysis Reveals Antibiotic-Susceptible Clones and Emerging Resistance in Neisseria gonorrhoeae in Saskatchewan, Canada. *Antimicrobial agents and chemotherapy* 2020; **64**(9).
25. Peng JP, Yin YP, Chen SC, et al. A Whole-genome Sequencing Analysis of Neisseria gonorrhoeae Isolates in China: An Observational Study. *EClinicalMedicine* 2019; **7**: 47-54.
26. Pinto M, Rodrigues JC, Matias R, et al. Fifteen years of a nationwide culture collection of Neisseria gonorrhoeae antimicrobial resistance in Portugal. *Eur J Clin Microbiol Infect Dis* 2020; **39**(9): 1761-70.
27. Reimche JL, Chivukula VL, Schmerer MW, et al. Genomic Analysis of the Predominant Strains and Antimicrobial Resistance Determinants Within 1479 Neisseria gonorrhoeae Isolates From the US Gonococcal Isolate Surveillance Project in 2018. *Sexually transmitted diseases* 2021; **48**(8s): S78-s87.
28. Ryan L, Golparian D, Fennelly N, et al. Antimicrobial resistance and molecular epidemiology using whole-genome sequencing of Neisseria gonorrhoeae in Ireland, 2014-2016: focus on extended-spectrum cephalosporins and azithromycin. *Eur J Clin Microbiol Infect Dis* 2018; **37**(9): 1661-72.
29. Sánchez-Busó L, Golparian D, Corander J, et al. The impact of antimicrobials on gonococcal evolution. *Nature Microbiology* 2019; **4**(11): 1941-50.
30. Thomas JC, Joseph SJ, Cartee JC, et al. Phylogenomic analysis reveals persistence of gonococcal strains with reduced-susceptibility to extended-spectrum cephalosporins and mosaic penA-34. *Nature communications* 2021; **12**(1): 3801.
31. Town K, Field N, Harris SR, et al. Phylogenomic analysis of Neisseria gonorrhoeae transmission to assess sexual mixing and HIV transmission risk in England: a cross-sectional, observational, whole-genome sequencing study. *The Lancet Infectious diseases* 2020; **20**(4): 478-86.
32. Jamoralin MC, Jr., Argimón S, Lagrada ML, et al. Genomic surveillance of Neisseria gonorrhoeae in the Philippines, 2013-2014. *Western Pac Surveill Response J* 2021; **12**(1): 17-25.
33. Williamson DA, Chow EPF, Gorrie CL, et al. Bridging of Neisseria gonorrhoeae lineages across sexual networks in the HIV pre-exposure prophylaxis era. *Nature communications* 2019; **10**(1): 3988.
34. Yahara K, Nakayama SI, Shimuta K, et al. Genomic surveillance of Neisseria gonorrhoeae to investigate the distribution and evolution of antimicrobial-resistance determinants and lineages. *Microb Genom* 2018; **4**(8).
35. Heng L. Aligning sequence reads, clone sequences and assembly contigs with BWA-MEM. *arXiv* 2013.

36. García-Alcalde F, Okonechnikov K, Carbonell J, et al. Qualimap: evaluating next-generation sequencing alignment data. *Bioinformatics* 2012; **28**(20): 2678-9.
37. Walker BJ, Abeel T, Shea T, et al. Pilon: an integrated tool for comprehensive microbial variant detection and genome assembly improvement. *PLoS One* 2014; **9**(11): e112963.
38. Bankevich A, Nurk S, Antipov D, et al. SPAdes: a new genome assembly algorithm and its applications to single-cell sequencing. *J Comput Biol* 2012; **19**(5): 455-77.
39. Seemann T. Prokka: rapid prokaryotic genome annotation. *Bioinformatics* 2014; **30**(14): 2068-9.
40. Croucher NJ, Page AJ, Connor TR, et al. Rapid phylogenetic analysis of large samples of recombinant bacterial whole genome sequences using Gubbins. *Nucleic acids research* 2015; **43**(3): e15.
41. Letunic I, Bork P. Interactive Tree Of Life (iTOL) v5: an online tool for phylogenetic tree display and annotation. *Nucleic acids research* 2021; **49**(W1): W293-w6.
42. Bushnell B, Rood J, Singer E. BBMerge - Accurate paired shotgun read merging via overlap. *PloS one* 2017; **12**(10): e0185056.
